# Cumulative Impacts of Diverse Land Uses in British Columbia, Canada: Application of the “EnviroScreen” Method

**DOI:** 10.3390/ijerph191811171

**Published:** 2022-09-06

**Authors:** Chris G. Buse, Aita Bezzola, Jordan Brubacher, Tim K. Takaro, Arthur L. Fredeen, Margot W. Parkes

**Affiliations:** 1 Faculty of Health Sciences, Simon Fraser University, Burnaby, BC V5A 1S6, Canada; 2 School of Health Sciences, University of Northern British Columbia, Prince George, BC V2N 4Z9, Canada; 3 Natural Resources and Environmental Studies Institute, University of Northern British Columbia, Prince George, BC V2N 4Z9, Canada

**Keywords:** health impacts of land use change, environmental health justice, cumulative impacts, CalEnviroScreen, BCEnviroScreen

## Abstract

(1) Objectives: Cumulative impacts refer to the legacies of land use decisions on environmental, community and health values. New integrative impact assessment tools are required to assess cumulative impacts on diverse values to meet sustainability goals in the 21st century. In this contribution, the CalEnviroScreen methodology-a screening tool capable of merging environmental, socioeconomic and health data-is applied to Local Health Areas in British Columbia, Canada. (2) Methods: The CalEnviroScreen is a method that standardizes environmental, socioeconomic and health data to depict an indicator’s percentile rank in the distribution of all units of analysis. The method combines indicators to measure four dimensions of pressure: environmental exposures, environmental effects, socioeconomic conditions, and sensitive populations (i.e., health outcomes). We create two versions of EnviroScreen: one following the CalEnviroScreen suite of indicators, and another that uses nuanced indicators to approximate the realities of industrial land uses present in British Columbia. BCEnviroScreen scores are plotted by race/ethnicity to understand potential racial inequities in cumulative exposures. (3) Results: The BCEnviroScreen has a greater likelihood of quantifying the cumulative impacts of diverse industries and land uses present across resource-dependent parts of the province, relative to the more urban-centric CalEnviroScreen indicator suite. Analyzing the distribution of BCEnviroScreen scores by race/ethnicity suggests that visible minority populations may be inequitably exposed to cumulative impacts in BC. (4) Conclusion: EnviroScreen tools hold significant potential to influence Canadian environmental health policy. This research demonstrates the applicability of the tool to British Columbia and other jurisdictions, illustrates how indicators can be tailored to better represent regional context, and shows how the tool can be used to screen for potential environmental health injustices.

## 1. Introduction

Land use change is increasingly recognized as an important driver of human health and well-being. Processes of urbanization, agricultural and other forms of resource development, and road construction and deforestation can alter habitats and increase the likelihood of human exposure to zoonotic and vector-borne diseases through direct transmission and ‘spill-over’ events [1,2]. Research also suggests that a variety of anthropogenic disturbances to land also can hold direct and indirect health impacts, whether from soil contamination [3,4], threats to ground [5] and surface water [6], or changes in air quality [7]. There is also a robust literature on the numerous exposure pathways of extractive industries impacts on human health [8]. Taken together, these effects can accumulate over time and space and leaving lasting legacies for human health and well-being.

A notable shortcoming of this literature is that it primarily traces biophysical and toxicological exposure pathways of land use change on human health. What is often missing is an accounting of the broader determinants of health in the assessment of accumulating and cumulative impacts of diverse land uses. Doing so would bring together the ecological and social determinants of health to yield a next generation of integrative impact assessments rooted in a framework of environmental justice [9]. Such assessments could assist land use planners and public health professionals promote human health and protect populations with higher exposure or lower capacity to adapt to changing environments.

This contribution presents a pilot of an integrative cumulative impacts screening method that merges environmental, community and health data into a regional assessment architecture in the context of British Columbia (BC), Canada. It deploys the CalEnviroScreen methodology-a relativistic spatial analysis tool and leading example of integrative impact assessment internationally. The tool enables users to depict where and who may be differentially exposed to cumulative impacts of diverse land uses. We first describe the methodological orientation and process employed to undertake our analysis, including establishing two treatments of the EnviroScreen—one fit to represent the CalEnviroScreen suite of indicators, and a second that is fit to better represent the land use realities of BC. It presents findings on land use pressures and their interface with environmental health justice concerns, and discusses opportunities for improving the method to understand cumulative impacts. Accordingly, the objectives of this study are three-fold:

1. Apply the CalEnviroScreen method to the Canadian context of BC to determine how well it approximates known landscape-level pressures.
2. Refine the indicators in the model by tailoring it more explicitly to the BC context to create a BCEnviroScreen tool.
3. Analyze BCEnviroScreen scores in relation to race/ethnicity to understand potential environmental justice implications of the analysis.

## 2. Background

‘Cumulative impacts’ are a long-standing concern in Canadian environmental science. For the purposes of this paper, they refer to the past, present and future interactions between diverse land uses (e.g., multiple forms of extractive industries and supporting infrastructure, land cover changes, climate impacts) and lasting legacies those interactions leave for environments, communities and health [10]. Classic examples of cumulative impacts include the impacts on water flow rates and quality, temperature, and fish populations of multiple run-of-river dams [11], or worsening air quality from multiple point sources of pollution in a single airshed [12]. At the individual level, a cumulative impact can be thought of as the cumulative dose of a point source exposure (e.g., a fire fighter is exposed to particulate matter at work, and also smokes cigarettes, compounding their exposure). It can also refer to multiple environmental exposures in an exposomic sense (e.g., any number of chemical exposures through air, food and water, lifestyle behaviours, extreme weather, etc., accumulated over the life course). At the community level, examples may include increased traffic congestion during the construction of a major project, and/or the strain on local community infrastructure and services from increased employment during a resource boom [13].

In Canada, cumulative impacts are primarily assessed as part of environmental impact assessment (EIA) processes at the provincial and federal levels. While the discourse of cumulative impacts has made important contributions to understanding how key economic drivers of the Canadian economy (e.g., forestry, oil and gas, mining) affect the integrity of ecosystems, there is increasing recognition that assessing cumulative impacts within the context of EIA is limited in scope.

EIAs tend to be focused on a single project’s footprint, which risks missing important transboundary impacts of pollution that ‘flow’ from a project or land use. Past research has argued this approach creates conditions for cumulative impacts to emerge due to the lack of consideration of interacting land uses [14]. Moreover, the recognition that ecosystems are the foundation for community economic and social development, and significant determinants of human health, necessitates an integrative focus on cumulative impacts [15,16,17].

Both the Impact Assessment Act, 2019 and the BC Environmental Assessment Office each recognize that major projects and associated land uses may cumulatively impact environmental, socio-economic, socio-cultural and human health conditions. While Health Impact Assessment has existed for decades as a tool to support EIA, it is still primarily focused at the level of a single project, and even understanding cumulative health effects is primarily considered in relation to facility-by-facility and chemical-by-chemical exposures [18,19], which may miss interactions between environmental exposures, environmental outcomes, and degrees of socioeconomic marginalization which interact to produce ill-health. This has led researchers to argue for incorporating psychosocial [20] and socio-economic determinants of health [21] into an understanding of cumulative impacts, and that cumulative impacts may unjustly impact marginalized populations by reflecting broader societal inequities such as environmental racism [22]. As a result, cumulative impacts are increasingly implicated in social license to operate, and land use planners, resource planners, and public health professionals require new tools to account for and remedy their impacts across environmental, community and health domains [10].

This is especially relevant for public health practitioners given that health tends to be underrepresented in EIA processes beyond workplace and toxicological exposures [8]. This creates an integrative and intersectoral challenge in supporting healthy public policy-to merge data, expertise and imperatives of multiple sectors and disciplines to understand the health implications of cumulative impacts. It also requires a whole-systems understanding of ‘impact’ and the need for intersectoral responses to adequately account for integrative dimensions of multiple land use values [23]. Indeed, a recent review of essential elements of impact assessment highlighted cumulative effects, sustainability and suitable regional/strategic assessments as leading praxis [24].

Fortunately, approaches that merge the imperatives of environmental, social and health impact assessment into a single architecture are being created by environmental justice advocates to support actions that protect marginalized populations and promote human health [9]. Examples include the US Environmental Protection Agency’s Environmental Justice Screening Tool [25] which has been applied locally vis-à-vis the CalEnviroScreen [26], the Washington Environmental Health Disparities Map [27], and the Chicago Cumulative Impacts Map [28], among others. To date, at least 17 states in the United States have developed mapping tools to explore cumulative environmental health effects in ways that inform land use decision-making [29]. However, no such tools have ever been applied to the Canadian context, and less attention has been given to the rural and remote and largely resource-dependent regions where natural resource extraction occurs. Addressing these issues may assist the intersectoral integration of health concerns into EIAs in Canada, and support public planning conversations more generally. Importantly, they may also draw attention to environmental health justice issues in Canada-the unfair and often avoidable ill-health outcomes that stem from environmental exposures [9,30].

## 3. Methods

This research trials an innovative cumulative impacts screening tool capable of integrating diverse forms of data to characterize environmental, community and health pressures in a Canadian context. We draw inspiration from the CalEnviroScreen tool, a relativistic analysis method that uses 20 indicators to approximate four domains of impact in the state of California: environmental exposures, environmental effects, socioeconomic marginalization and sensitive populations. The method works by creating a percentile rank for each indicator to develop a standardized and composite score for the four domains above, and to then scale them into a singular measure that represents cumulative impacts for a landscape unit (e.g., a census tract in the case of CalEnviroScreen), in relation to all other units in the sample.

### 3.1. Study Context: British Columbia, Canada

**Figure 1:**
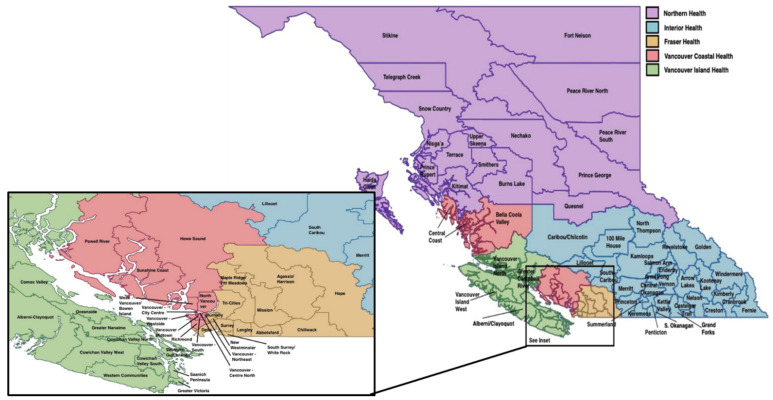
The five regional health authorities and corresponding Local Health Areas in British Columbia, Canada.

BC is Canada’s western-most province, comprising approximately 944,735 square kilometers, and in 2020 was home to a population of 5.1 million people. The majority of the population lives in the densely populated southwest coastal region that comprises Metro Vancouver and its surrounding suburbs, and the south Vancouver Island area of Metro Victoria. The province is largely located on the un-ceded and traditional territories of numerous Indigenous nations and communities, with the exception of the northeast region which is part of Treaty 8 territory signed in 1899. The majority of the province’s land area is rural and remote (particularly the Northern Health and Interior Health regions, see Figure 1) with long histories of resource extraction and industrial land uses from forestry, industrial agriculture and livestock, mining, oil and gas extraction, hydroelectric projects, and other forms of renewable power generation. The province is divided into 5 regional health authorities comprised of 89 Local Health Areas (LHAs) (see Figure 1) that are the lowest order of geographical unit that health information is able to be publicly accessed across the province. For this reason, LHAs serve as the unit of analysis in this study.

### 3.2. EnviroScreen Indicators and Score Computation

Two versions of a EnviroScreen were created utilizing BC data. Version 1 (herein ‘CalEnviroScreen’) used indicators that followed the CalEnviroScreen 3.0 indicator suite [26] as closely as possible. Version 2 (herein ‘BCEnviroScreen’) expanded on these indicators based on the study team’s multi-year collaboration on cumulative effects in Northern BC, which was deepened by insights through their engagement with the Environment, Community, Health Observatory Network-a pan-Canadian research community interested in cumulative impacts. This included the identification and sourcing of publicly available data that better reflects economic, social and environmental conditions in BC (see Table 1 and Table 2).

For the analysis and creation of composite scores for each domain of impact (e.g., environmental effects, environmental exposures, socioeconomic conditions, sensitive populations), each version followed the CalEnviroScreen methodology. First, a ranked percentile calculation was applied to each LHA for every indicator in our sample to transform raw values into a standardized, relative score (e.g., all scores are presented in relation to all other scores for each geographic unit (LHAs) within our sample). This enables the percentile ranking (on a scale of 0–100) of a given LHA to be utilized in place of the raw value for any given indicator, therefore enabling diverse data points to be indexed. CalEnviroScreen is agnostic regarding data distribution. Given that some data points may not be normally distributed, normalized transformations (e.g., z-scores) are not entirely appropriate. Instead, the model represents each geographic unit in relation to the percentage of other geographic units that are above or below it in the total distribution.

The percentile rankings of all indicators in each of the four indicator categories (i.e., environmental exposures, environmental effects, socioeconomic conditions and sensitive populations) are then added together, and averaged for each LHA to compute a final score of impact for each of the four categories. Next, a “population characteristics score” is calculated as an average of the socioeconomic conditions and sensitive population scores, and a pollution burden score was calculated as an average of environmental exposure and environmental effects, where environmental effects are weighted by one half to account for the uncertainty of relationships between exposures and effects. The population characteristics and pollution burden scores are then scaled in relation to the largest LHA value in the sample to produce a relativistic score:

$$\left(\frac{Individual\;Local\;Health\;Area\;Score}{Largest\;Local\;Health\;Area\;Score}\times 10\right)$$

Finally, population characteristics and pollution scores are multiplied to produce a final EnviroScreen Score out of 100, with the multiplication representing the interaction between socioeconomic and ecological systems. In the following section, all scores across each treatment are mapped, and a percent change in scores is calculated to show the degree to which LHA scores changed between indicator treatments. All transformations were conducted using Microsoft Excel, and data was then spatially mapped using ArcGIS Version 10.

Indicators with values of 0 (e.g., no forestry sawmills in the area) were not used to calculate percentiles but were assigned a value of 0 and included in final group scoring. Missing data (driven by low-count census data) were not used to calculate the percentiles and not included in the final group score. 

## 4. Results

Our application of the CalEnviroScreen method to the province of BC resulted in comparative analyses between two treatments of the screening method. The first version approximated the indicators of the CalEnviroScreen as closely as possible, whereas the second version—the BCEnviroScreen—utilized indicators that were more attuned to the nuanced dynamics of industrial disturbance and anthropogenic land use present across BC. Results of applying the EnviroScreen methodology across both treatments are presented, highlighting similarities and differences between the models.

### 4.1. CalEnviroScreen Pollution Burden (Version 1) vs. BCEnviroScreen Pollution Burden (Version 2)

**Figure 2:**
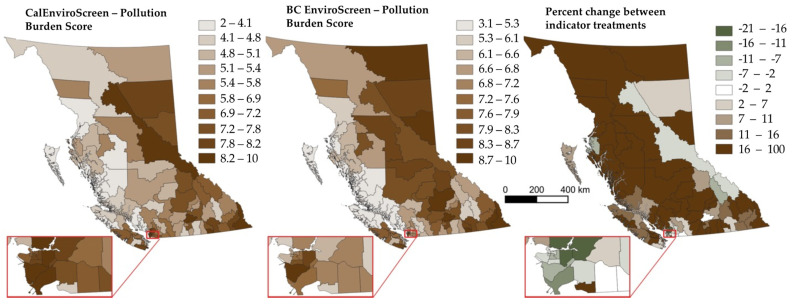
Comparing two treatments of EnviroScreen indicators on ‘pollution burden’ scores in British Columbia, Canada.

Pollution burden scores reflect the combined impacts of environmental exposures and effects in a single measure. In the CalEnviroScreen indicator suite, the pollution burden scores tend to be higher and clustered in the most populated parts of the province including the Capital Region of Victoria and its surrounding LHAs, Metro Vancouver LHAs (notably Richmond, Burnaby and Northern Vancouver), the Central Interior (which includes the City of Kelowna) and Prince George LHA (see Figure 2). However, the CalEnviroScreen representation of pollution burden appears to underrepresent the influence of oil and gas exploration and development in the northeast (i.e., Fort Nelson and South Peace LHAs), and long standing mining and forestry operations throughout LHAs across the central interior region of the province.

The inclusion of industrial land uses more relevant to BC better reflects the land use realities that are present across more rural, remote and resource dependent regions where the vast majority of the province’s natural resources are extracted. In the BCEnviroScreen, Richmond LHA in the Metro Vancouver region is still among the highest scoring LHAs; however, almost all LHAs across the central interior, the northwest and the northeast (with the exception of the North Peace LHA) show higher pollution burden scores than the CalEnviroScreen treatment. These scores reflect the literature base on the scale of ecological degradation, particularly in the northeast of the province which is host to overlapping industries of forestry, oil and gas exploration and mining operations [23,31,32]. Fernie is the highest scoring LHA in the BCEnviroScreen, reflecting its longstanding history of mining, forestry and tourism.

In both versions, measurements of air and water pollution (environmental exposures) differentially affect the southeast area of the province, and more densely populated areas of Vancouver, Victoria and the central Okanagan. With the inclusion of future climate data, northern BC LHAs score higher given the faster rate of warming being experienced relative to coastal and southern regions of the province. The inclusion of climate data is an increasingly important function of environmental exposures in BC given recent risks associated with flooding [33], wildfires [34] and extreme heat [35]. Notably, the inclusion of these and other variables in the BCEnviroScreen perhaps requires a change in moniker from a ‘pollution burden’ score to something that approximates a cumulative measure of ‘landscape burden’.

Each treatment of the EnviroScreen method showed evidence of spatial autocorrelation (CalEnviroScreen Treatment: Moran’s I z-score = 9.6, *p* < 0.001; BCEnviroScreen Treatment: Moran’s I z-score = 4.6, *p* < 0.001), where higher scoring regions tended to border higher scoring regions (and vice versa). This makes intuitive sense insofar as most resource operations require supporting infrastructure that transcends socially defined jurisdictional boundaries. The highest and lowest pollution burden scores across treatments can be found in Supplemental Table S1.

### 4.2. Population Characteristics Scores

**Figure 3:**
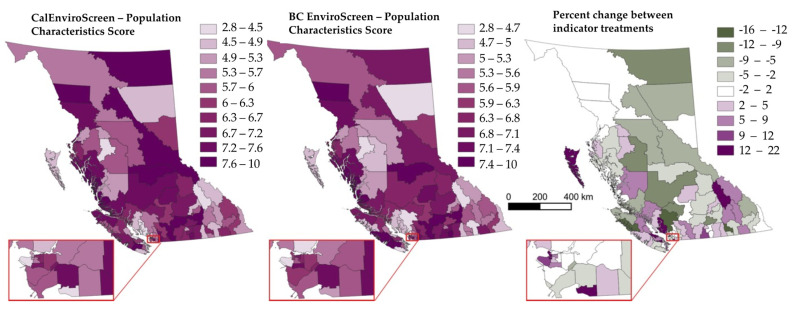
Comparing two treatments of EnviroScreen indicators on ‘population characteristics’ scores in British Columbia, Canada.

Population characteristics scores (an expression of socioeconomic marginalization and population health) only differed in terms of the inclusion of diabetes, based on its recognition as a health outcome of significance for Indigenous populations [36]. In each version, there is no evidence of spatial clustering, but both models project higher values for the population characteristics among coastal LHAs (see Figure 3). The inclusion of diabetes led to increases in Enviroscreen scores in BCEnviroScreen for Metro Vancouver, the southern interior, and the Kootenay region in the southwest of the province. The highest and lowest population characteristics scores across treatments can be found in Supplemental Table S2.

### 4.3. CalEnviroScreen vs. BCEnviroScreen Scores

**Figure 4:**
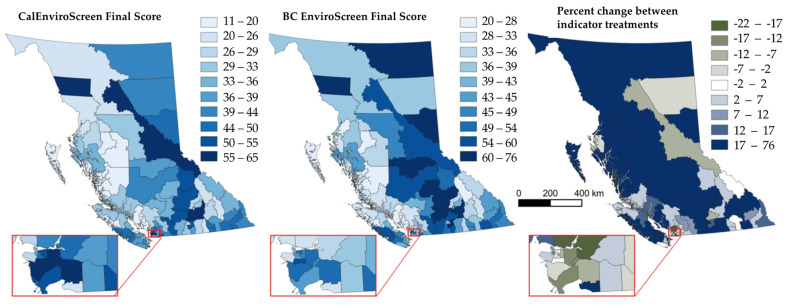
Comparing two treatments of EnviroScreen indicators on overall EnviroScreen scores in British Columbia, Canada.

The two applications of the EnviroScreen methodology led to different results in terms of overall EnviroScreen scores (see Figure 4). EnviroScreen scores are intended to reflect the confluence of environmental, socioeconomic and health impacts, and therefore could be thought of as the integration of diverse land use values that approximate a measure of cumulative impacts at a regional scale. An important feature of the EnviroScreen is that as a screening tool, results can be put into context of broader justice concerns playing out at a regional level (e.g., Indigenous Treaty Rights infringement related to multiple land uses) and can share data that reflects cumulative pressures on certain places.

In the CalEnviroScreen treatment, the urban areas of Richmond, Burnaby and Surrey are in the highest decile of EnviroScreen scores. The Central Okanagan has the highest CalEnviroScreen score of all LHAs, followed by Prince George, and Telegraph Creek. Coastal LHAs and those located in the southeast of the province score lower than interior LHAs, or those located near major population centres of Vancouver and Victoria. CalEnviroScreen scores are spatially autocorrelated more than would be expected by chance alone, suggesting that LHAs with similar cumulative pressures tend to border similarly scoring regions (Moran’s I = 6.2, *p* < 0.001).

BCEnviroScreen produces a different set of results. The previous high-scoring urban LHA scores are lower relative to the entire sample, and while there is also evidence of spatial autocorrelation overall (Moran’s I = 2.8, *p* < 0.01), spatial clustering is strongest across the central interior and the northeast of the province. These results suggest that CalEnviroScreen indicators may be more urban-centric, and when applied to the BC context, may not adequately reflect the types of past and present industrial exposures that exist beyond urban areas. However, BCEnviroScreen appears to more accurately reflect the types of land use that are typically found across BC’s rural, remote and resource dependent regions, and may better represent cumulative impacts in these unique contexts. The highest and lowest EnviroScreen scores across treatments can be found in Supplemental Table S3.

### 4.4. BCEnviroScreen Scores Compared against Population Proportions by Race/Ethnicity

The EnviroScreen maps do not show disparities within and between population groups, but rather geographically depict where cumulative environmental, socioeconomic and health pressures are greatest. One of the unique aspects of the EnviroScreen method is that it enables users to plot the distribution of scores in relation to population demographics to gain a sense of differential exposure to environmental, community and health pressures within unique population groups across the entire sample. This enables researchers to understand elements of environmental justice related to cumulative impacts.

**Figure 5:**
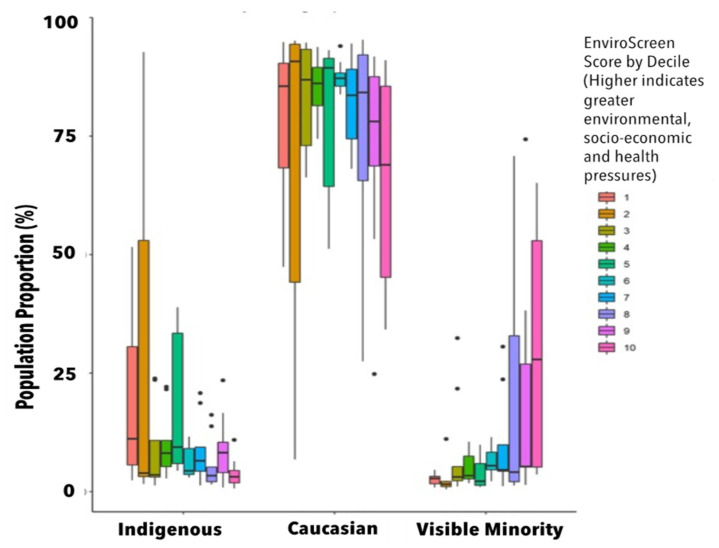
Within group racial/ethnic proportions by decile of BCEnviroScreen score, expressed as a proportion of all Local Health Areas in British Columbia, Canada.

Figure 5 displays ten deciles of BCEnviroScreen scores by population proportions of selected races/ethnicities, where the dots are outlying values. The results suggest that the visible minority-identifying populations median population proportion is greatest for the highest scoring EnviroScreen decile, and that the interquartile range is largest for the three highest deciles of impact. This demonstrates differential exposure of visible minority-identifying populations to cumulative impacts as represented by the BCEnviroScreen score. It is also in stark contrast to the uniform distribution of Caucasian-identifying population proportions across all deciles of EnviroScreen scores, which typically range between 70–90%, irrespective of EnviroScreen decile. Conversely, within Indigenous-identifying populations across all LHAs, the median population proportion was greatest for the lowest decile of EnviroScreen scores.

## 5. Discussion

Given the increasing relevance of ‘cumulative impacts’ in Canadian environmental science, and rising interest in the environmental justice discourse in Canada [30,37], new tools are required to integrate diverse land use values to account for and remedy them [10]. To the authors’ knowledge, this is the first ever application of an integrated, geospatial, environmental health screening tool to be applied in the Canadian context. By tailoring a new indicator suite based on publicly available data, the BCEnviroScreen shows how environmental health inequalities tools developed in the United States can be applied to the Canadian context in ways that account for local and regional land uses. The CalEnviroScreen treatment more closely approximated the original CalEnviroScreen indicators and better reflects exposures in urban environments, whereas the expanded suite of indicators in the BCEnviroScreen are more applicable to the realities of land uses in rural, remote and resource-dependent communities. This suggests that different treatments of the tool may be needed for urban-only versus rural-only comparisons.

The approach also provides evidence that visible minority populations may be differentially exposed to cumulative environmental, community and health pressures, insofar as greater population proportions live in LHAs with higher EnviroScreen scores. This corroborates with evidence from California [38], and is suggestive of environmental injustices which may stem from the unfair and largely avoidable exposure to environmental and social harms which may drive disparities in ill-health health and socioeconomic status [39]. These injustices may also reflect evidence of environmental racism, although further research is required to fully understand the structural pathways by which racialized populations may be impacted by changes in land use over time.

Further, Indigenous population proportions are highest in LHAs with lower deciles of EnviroScreen scores. Many Indigenous communities in BC reside in rural, remote, and relatively pristine environments which may account for lower scores. However, it may also be that in a province with majority unceded territories and limited coverage by formal treaties (notwithstanding Treaty 8 in the northeast), that this is a story of resilience and Indigenous stewardship over the land. This is supportive of past research showing Indigenous-managed lands have as high or higher levels of biodiversity relative to protected areas [40,41]. These dynamics require further research to understand, especially given that Indigenous populations have the heaviest burdens of ill health in Canada and internationally [42], and important health impacts may go unmeasured as related to resource development and land use change [43]. Future analysis could examine other forms of intersecting identity factors to understand the distribution of EnviroScreen scores across age, gender and socioeconomic class to further understand how issues of systemic oppression may unfairly expose certain populations to higher cumulative pressures. Finer resolution of environmental pressures stratified by race/ethnicity could further validate these findings.

The results also demonstrate how the EnviroScreen method is one of a few promising approaches that can spatially depict the notion of ‘cumulative impacts’ through integrative assessment of environment, community and health values and diverse data sets. As a result, the methodology holds potential to drive decision-making processes in ways that recognize the importance of environmental change, socioeconomic marginalization, and human health. Additionally, the EnviroScreen could support the development of educational programs to build community awareness and engagement, pre-construction assessments that could help reduce impacts in the first place, and the on-going identification and monitoring of impacted areas and their interactions with vulnerable populations. This makes these analytic methods an ideal research tool to support impact assessment processes, land and resource planning, and population-level health assessment.

Given these strengths, we see significant potential in developing other applications of these tools across Canadian provinces, and there are several pilots already underway for Alberta, New Brunswick and Ontario by ECHO Network researchers and trainees, including transforming this analysis into interactive, on-line tools. Future work should also explore the potential to apply this methodology at discrete levels of analysis that better match unique ecological units such as watersheds, airsheds, or other unique geographies to better understand how industrial pressures may interact with socioeconomic issues in the production of health and well-being in bounded geo-physical units of analysis [38,44].

Despite its strengths, the EnviroScreen method is not a panacea for ‘solving’ challenges associated with what is referred to as the “integration imperative” [10]-merging environmental, community and health data into a common decision-making framework to redress cumulative impacts. There are several limitations of the tool that could be rectified through future research, which have not been adequately addressed in the literature.

First, EnviroScreen scores are relative and do not communicate actual risk, but rather screen issues relevant to environmental justice considerations when considering the siting of major projects or undesirable land uses. This makes it an ideal tool for supporting regional and/or strategic environmental assessment rather than the assessment of cumulative effects of a single project. As a result, it does not necessarily depict causal or dose-response relationships between exposures and outcomes. Future research could validate these models using factor analytic methods, and examine ‘pathways of effect’ across the sub-components of the EnviroScreen scores to determine the relative contribution and influence on human health outcomes.

Second, the percentile rank feature is ideal for dealing with non-normal data, but as a result of its calculation, can magnify the appearance of risk if the spread in distribution for a given indicator may be relatively small when expressed as an absolute value. Nonetheless, the scores do benchmark geospatial units to enable a strong sense of comparison. This can encourage decision-makers in neighboring jurisdictions to examine policies, plans or programs that are working to produce relatively lower scores, creating an opportunity for innovative pilot interventions or programs that seek to improve many of the indicators included in the model.

Third, a key element of cumulative impacts is the accumulation of impacts across time in addition to space [45]. While the analysis conducted here can be thought of as a snapshot of historical land use decisions and/or the accumulation of environmental, community and health issues over time, it does not yet include historical comparison. This is partly due to the fact that historical data for many included indicators (notwithstanding census data) are often missing, or incompatible based on data collection, coverage, reliability and consistency of measures across all regions of the province. Utilizing these data to benchmark future changes in scores over time would significantly enhance the value and utility of the BCEnviroScreen moving forward.

Fourth, there are limitations to the data, and a degree of uncertainty in each included indicator. The EnviroScreen method requires analysts to balance data quality and availability at each landscape unit of analysis. Many environmental concerns are best measured within specific ecosystems, biomes or even watersheds that do not align nicely with pre-defined jurisdictions such as census dissemination areas or LHAs. Future research should seek to examine the ability to improve the granularity of environmental data to a landscape unit that is commensurate with appropriate monitoring to reduce degrees of uncertainty [46]. The major challenge for this type of analysis is that the availability of health data is often at a much less granular level given privacy concerns associated with certain conditions and diseases, which makes the reporting of health concerns at a smaller order spatial scale a challenge. Moreover, we purposefully utilized publicly accessible data that we know is regularly collected to support longitudinal monitoring, rather than opt for indicators that were part of ‘one-off’ data collection processes, to reduce the burden of updating the models. All these issues raise the question of what is the ‘right’ resolution for measuring large land use changes, anthropogenic forcings on landscapes and their cumulative impacts. These questions are best posed in broad consultation with potential knowledge users of the tool.

Finally, the EnviroScreen method is clearly rooted in a deficit model of understanding the impacts of environmental change and the social determinants on health. While this can be helpful for prioritizing areas that require intersectoral action to redress cumulative impacts-such as the creation of conservation initiatives in LHAs with high pollution burden scores-it also risks stigmatizing areas based on their scores, or encouraging them to be viewed as so-called ‘sacrifice zones’ for future development [47]. Future work to understand a series of assets-based indicators may help to promote resilience to ecological and socioeconomic shocks [48,49]. Examples could include the proportion of communities that have developed climate action plans or the proportion of environmentally-remediated lands. This could be achieved through merging quantitative frameworks with qualitative and strength-based approaches to understanding and responding to cumulative impacts [15,50].

## 6. Conclusions

A persistent challenge in the realm of cumulative impacts—whether environmental, socioeconomic or health-related—is that no sector can effectively govern them alone. This is a perpetual challenge for public health practitioners seeking to redress health inequalities and promote health from local to regional scales, but particularly for those working in rural, remote and resource-dependent contexts. Building on long-standing calls for intersectoral action to promote health, integrative tools that merge diverse forms of data and information are required if public health is to meaningfully engage with wide-ranging impacts of environmental change on human health and socio-economic development. This is especially true in the case of resource governance, where EnviroScreen tools offer potential to support regional and strategic environmental assessment of cumulative impacts, as well as health impact assessment procedures. Not only has this research demonstrated the applicability of this tool to the Canadian context, but it has demonstrated how indicator suites can be tailored to match unique regional context(s), and how the tool can be utilized to screen for potential environmental health injustices that warrant further investigation. This is especially the case of utilizing the tool to screen for potential environmental justice issues present in Canada and beyond.

In spite of its limitations, the unique regional and comparative assessment techniques enables an accounting of diverse forms of past, present and future data to broaden the imperatives of natural resource governance and land use decision-making. By creating outputs that are easily digestible by diverse audiences, these tools hold potential to engage stakeholders and Indigenous rightsholders in land use decision-making processes in ways that capture regional environmental, community and health impacts as opposed to measuring cumulative impacts at the level of a singular project. It also can draw attention to the realities of differential exposure to environmental challenges and cumulative effects in rural and remote settings.

## Figures and Tables

**Table 1:** Indicator definitions and data sources.

Indicator	Definition	Data Source
Education	Percent of the population (aged 25 to 64 years in private households) with no certificate, diploma or degree. Based on 25% sample data, aggregated from 2016 Dissemination Area data.	Statistics Canada, 2016
Employment	Percentage of labour force (over 15 years) which is unemployed. Aggregated from 2016 Dissemination Area data.	
Low income	Percentage of population (aged 18 to 64 in private households to whom low-income concepts are applicable) in low income based on the Low-income measure, after tax (LIM-AT). Aggregated from 2016 Dissemination Area data.	
Linguistic isolation	Percentage of population (excluding institutional residents) with no knowledge of French or English. Aggregated from 2016 Dissemination Area data.	
Housing burdened	Percentage of population (owner and tenant households with total income greater than zero, in non-farm, non-reserve private dwellings by shelter-cost-to-income ratio) which spend 30% or more of income on shelter costs. Based on 25% sample data, aggregated from 2016 Dissemination Area data.	
Incidence of COPD	Chronic obstructive pulmonary disease (COPD) age standardized incidence rate (per 1000) 2015–2016	Provincial Health Service Authority, 2020
Hypertension	Hypertension (high blood pressure) age standardized incidence rate (per 1000) 2015/16	
Low-birth weight	Low birth weight rate (per 1000 live births) 2011–2015.	
All causes of cancer	All-cause cancer incident cases (all ages-total) (count) (2008–2012 Average)/2011 population	
Diabetes mellitus	Diabetes mellitus (DM) age standardized incidence rate (per 1000) 2015/16,	
Environmental remediation sites	Count of known and potentially contaminated properties in British Columbia. A site is contaminated if its land, water and/or sediment are unsuitable for particular uses from waste that exceeds environmental quality standards.	British Columbia Ministry of Environment and Climate Change Strategy-Environmental Emergencies and Land Remediation, 2020
Linear footprint	The total length of human disturbance lines for each LHA (km) divided by the LHA area (km2). Created using forest tenure road section lines, railway track line, Digital Road Atlas, BC transmission lines, Pipeline segments, Transmountain pipeline, Geophysical plans, and Geophysical lines. Forest service roads located within 1km of roads from the Digital Road Atlas, geophysical plans located within 1km of geophysical lines, Geophysical lines that were labelled as ‘handcut’, and ‘aeromagnetic’ were removed to avoid over-representing disturbance.	British Columbia Ministry of Forests, Lands, Natural Resource Operations and Rural Development-Forest Tenures, 2018; Ministry of Forests, Lands, Natural Resource Operations and Rural Development-GeoBC, n.d.-a; Ministry of Forests, Lands, Natural Resource Operations and Rural Development-GeoBC, n.d.-b; Ministry of Forests, Lands, Natural Resource Operations and Rural Development-GeoBC, n.d.-c; BC Oil and Gas Commission, n.d.-a; BC Oil and Gas Commission, n.d.-b; BC Oil and Gas Commission, n.d.-c; Wilderness Committee, n.d.
Disturbed Land	Percent of LHA land-base not considered Intact Forest Land using data from 2016	Potapov et al., 2008
Forestry Mills	Count of mill facilities per LHA	Natural Resources Canada Strategic Policy and Results Sector, 2020
Smelters and refineries	Count of smelters and refineries per LHA	Natural Resources Canada Lands and Minerals Sector, 2020
Mines	Count of producing mines per LHA	
Oil and gas sites	Count of oil and gas fields per LHA	
Hazardous waste facilities	Count of hazardous waste facilities per LHA	British Columbia Ministry of Jobs, Economic Development and Competitiveness-International Marketing, 2020
Wildfire burn area-10 year	The percent of LHA’s burned by wildfires from 2010 to 2019	British Columbia Ministry of Forests, Lands, Natural Resource Operations and Rural Development-BC Wildfire Service, 2020
PM2.5	The annual mean concentration of PM 2.5 in 2012 for every LHA based on the postal code unit. The calculation was made by assigning each postal code centroid to the LHA it fell within and then calculating a mean of those values.	CANUE-The Canadian Urban Environmental Health Research Consortium, 2017b
Ozone	The annual mean concentration of O_3_ in 2015 for every LHA based on the postal code unit. The calculation was made by assigning each postal code centroid to the LHA it fell within and then calculating a mean of those values.	CANUE-The Canadian Urban Environmental Health Research Consortium, 2017a
Traffic density	Annual average daily traffic counts attributed to LHA’s from a Census Division level	BC Ministry of Transportation, 2018
EMS water quality exceedance	Percent of Environmental Monitoring System (EMS) sample locations in each LHA with an exceedance of BC Source Water Quality Guidelines for Total Lead (>0.005 mg/L), *E*. *coli* (>10/100 mL), NO_3_ dissolved (>45 mg/L), Mercury-all measures (>0.001 mg/L), Total Phosphorus (0.01 mg/L), Total Organic Carbon (4 mg/L)	British Columbia Ministry of Environment and Climate Change Strategy-Knowledge Management, 2020
Future temperature	Future annual temperature change in degrees Celsius (2010–2039 relative to 1961–1990 baseline) attributed to LHA’s from a Census Division level	Pacific Climate Impacts Consortium, 2020
Future precipitation	Future annual precipitation change as a percent (2010–2039 relative to 1961–1990 baseline) attributed to LHA’s from a Census Division level	

**Table 2:** Indicator comparison between Version 1 (CalEnviroScreen) and Version 2 (BCEnviroScreen) of the EnviroScreen methodology applied to British Columbia, Canada.

		CalEnviroScreen (Version 1)	BCEnviroScreen (Version 2)
Socioeconomic Factors	% < HS education	x	x
	% Unemployed	x	x
	% Low income	x	x
	% Linguistic isolation	x	x
	% Housing burdened	x	x
Sensitive Population	Incidence of COPD	x	x
	Hypertension	x	x
	Low–birth weight	x	x
	All causes of cancer	x	x
	Diabetes mellitus		x
Environmental Effects	Environmental remediation sites	x	x
	Linear footprint	x	x
	Disturbed Land (2016)		x
	Smelters		x
	Forestry Mills		x
	Mines		x
	Oil and gas sites		x
	Hazardous waste facilities	x	x
	Wildfire burn area-10 year		x
Environmental Exposure	Ozone	x	x
	PM2.5	x	x
	Traffic density	x	x
	EMS water quality exceedance-mercury	x	x
	EMS water quality exceedance-lead	x	x
	EMS water quality exceedance-phosphorus	x	x
	EMS water quality exceedance-nitrate	x	x
	EMS water quality exceedance-TOC (carbon)	x	x
	EMS water quality exceedance-*E. coli*	x	x
	Future temperature		x
	Future precipitation		x

## Data Availability

All data and materials are available upon request.

## Supplementary Materials

The following supporting information can be downloaded at: https://www.mdpi.com/article/10.3390/ijerph191811171/s1, Table S1: Highest and Lowest LHA Pollution Burden Scores from two treatments of the EnviroScreen Methodology in British Columbia, Canada; Table S2: Highest and Lowest LHA Population Characteristics Scores from two treatments of the EnviroScreen Methodology in British Columbia, Canada; Table S3: Highest and Lowest CalEnviroScreen and BCEnviroScreen Scores from two Applications of the EnviroScreen Methodology in British Columbia, Canada. References [51,52,53,54,55,56,57,58,59,60,61,62,63,64,65,66,67,68,69,70,71,72] are cited in the supplementary materials
