# Supplementary material for: Cumulative Impacts of Diverse Land Uses in British Columbia, Canada: Application of the “EnviroScreen” Method
Source: Int J Environ Res Public Health. 2022 Sep 6;19(18):11171. doi: 10.3390/ijerph191811171 (PMC9517321; doi:10.3390/ijerph191811171)
Supplement: Supplementary file 1 [file ijerph-19-11171-s001.zip › ijerph-1878734-Supplemental File.pdf]

Due to space limitations, we have created this supplemental file that includes two points of interest for the readers: 1) tables that illustrate the five highest and lowest scoring Local Health Areas for each index presented in the manuscript across both treatments, and 2) a full accounting of the sources for included data.

Highest and Lowest-scoring LHAs by EnviroScreen Treatment

Due to space limitations in the body of the accompanying manuscript, we are unable to provide a deep description of key changes to the highest and lowest scoring LHAs across both treatments of the EnviroScreen. Supplemental Tables S1, S2 and S3 report on the five highest and lowest scoring LHAs in terms of pollution burden, population characteristics and EnviroScreen scores by treatment. The results below coincide with the more general description of percent changes to overall scores which is more fulsomely explained in the main body text of the manuscript.

Table S1. Highest and Lowest LHA Pollution Burden Scores from two treatments of the EnviroScreen Methodology in British Columbia, Canada

**CalEnviroScreen – HIGHEST SCORE**		**BCEnviroScreen – HIGHEST SCORE**	
**LHA**	**Pollution Burden Score**	**LHA**	**Pollution Burden Score**
Burnaby	10	Fernie	10
Richmond	9.79	Peace River South	9.77
Central Okanagan	9.46	Kamloops	9.57
Greater Victoria	9.14	Central Okanagan	9.56
North Vancouver	8.84	Trail	9.01
**CalEnviroScreen – LOWEST SCORE**		**BCEnviroScreen – LOWEST SCORE**	
Central Coast	1.95	Central Coast	3.12
Powell River	2.34	Powell River	3.88
Bella Coola Valley	2.87	Haida Gwaii	4.05
Burns Lake	3.33	Sunshine Coast	3.70
Vancouver Island West	3.56	Bella Coola Valley	4.62

Table S2. Highest and Lowest LHA Population Characteristics Scores from two treatments of the EnviroScreen Methodology in British Columbia, Canada

**CalEnviroScreen – HIGHEST SCORES**		**BCEnviroScreen – HIGHEST SCORES**	
**LHA**	**Population Characteristics Score**	**LHA**	**Population Characteristics Score**
Telegraph Creek	10	Telegraph Creek	10
Central Coast	9.89	Central Coast	9.93
Merritt	9.70	Merritt	9.29
Cowichan Valley	8.49	Hope	8.87
Hope	8.42	Cowichan Valley	8.11
**VERSION 1 – LOWEST SCORES**		**VERSION 2 – LOWEST SCORES**	
Smithers	2.77	Smithers	2.83
Revelstoke	3.50	Southern Gulf Islands	3.98
Southern Gulf Islands	3.73	Revelstoke	4.03
Oceanside	3.99	Nelson	4.34
Vancouver Island West	4.08	Peace River North	4.41

Table S3. Highest and Lowest CalEnviroScreen and BCEnviroScreen Scores from two Applications of the EnviroScreen Methodology in British Columbia, Canada

**CalEnviroScreen – HIGHEST SCORES**		**BCEnviroScreen – HIGHEST SCORES**	
**LHA**	**CalEnviroScreen Score**	**LHA**	**BCEnviroScreen Score**
Central Okanagan	65.24	Merritt	75.9
Prince George	62.99	Telegraph Creek	74.45
Burnaby	62.62	Quesnel	67.19
Vernon	59.86	Kamloops	65.09
Telegraph Creek	57.85	Fort Nelson	63.23
**CalEnviroScreen – LOWEST SCORES**		**BCEnviroScreen – LOWEST SCORES**	
Powell River	11.30	Powell River	19.52
Bella Coola Valley	14.13	Haida Gwaii	20.3
Smithers	15.58	Smithers	22.47
Sunshine Coast	15.79	Sunshine Coast	23.30
Haida Gwaii	16.30	Southern Gulf Islands	23.92

Supplementary Data References

BC Oil and Gas Commission. (n.d.-a). *Geophysical Lines (Permitted)* [Data Warehouse]. BC Oil and Gas Commission Open Data Portal. Retrieved September 23, 2020, from https://data-bcogc.opendata.arcgis.com/datasets/bd0a685c1f614b4b89ace6564e5e3cc4_0

BC Oil and Gas Commission. (n.d.-b). *Geophysical Plans (1996-2004)* [Data Warehouse]. BC Oil and Gas Commission Open Data Portal. Retrieved September 23, 2020, from https://data-bcogc.opendata.arcgis.com/datasets/81d619920b6848e9a3f0f0201d126cae_0

BC Oil and Gas Commission. (n.d.-c). *Pipeline Segments (Permitted)* [Data Warehouse]. BC Oil and Gas Commission Open Data Portal. Retrieved September 23, 2020, from https://data-bcogc.opendata.arcgis.com/datasets/359b7e14fafa4abc84ff873bc55015fb_0

British Columbia Ministry of Environment and Climate Change Strategy. (2020). *B.C. Source Drinking Water Quality Guidelines: Guideline Summary.* (WQG-01; Water Quality GuidelineSeries). Province of British Columbia. https://www2.gov.bc.ca/assets/gov/environment/air-land-water/water/waterquality/water-quality-guidelines/approved-wqgs/drinking-water-and-recreation/source_drinking_water_quality_guidelines_bcenv.pdf

British Columbia Ministry of Environment and Climate Change Strategy - Environmental Emergencies and Land Remediation. (2020). *Environmental Remediation Sites* [Data Warehouse]. BC Data Catalogue. https://catalogue.data.gov.bc.ca/dataset/environmental-remediation-sites

British Columbia Ministry of Environment and Climate Change Strategy - Knowledge Management. (2020). *BC Environmental Monitoring System Results—4 Year Current Results* [Data Warehouse]. BC Data Catalogue. https://catalogue.data.gov.bc.ca/dataset/949f2233-9612-4b06-92a9-903e817da659

British Columbia Ministry of Forests, Lands, Natural Resource Operations and Rural Development - BC Wildfire Service. (2020). *Fire Perimeters—Historical* [Data Warehouse]. BC Data Catalogue. https://catalogue.data.gov.bc.ca/dataset/fire-perimeters-historical#edc-pow

British Columbia Ministry of Forests, Lands, Natural Resource Operations and Rural Development - Forest Tenures. (2018). *Forest Tenure Road Section Lines—Data Catalogue* [Data Warehouse]. BC Data Catalogue. https://catalogue.data.gov.bc.ca/dataset/forest-tenure-road-section-lines

British Columbia Ministry of Health - Health Sector Information Analysis and Reporting. (2020). *Local Health Area Boundaries* [Data Warehouse]. BC Data Catalogue. https://catalogue.data.gov.bc.ca/dataset/local-health-area-boundaries

British Columbia Ministry of Jobs, Economic Development and Competitiveness - International Marketing. (2020). *Hazardous waste facilities* [Data Warehouse]. BC Data Catalogue. https://catalogue.data.gov.bc.ca/dataset/hazardous-waste-facilities

CANUE - The Canadian Urban Environmental Health Research Consortium. (2017a). *Air Quality—Fine Particulate Matter (PM2.5) (Annual Dataset)* [Data Warehouse]. CANUE - The Canadian Urban Environmental Health Research Consortium. https://www.canuedata.ca/canue/metadata.php

CANUE - The Canadian Urban Environmental Health Research Consortium. (2017b). *Air Quality—Ozone (Annual Dataset)* [Data Warehouse]. CANUE - The Canadian Urban Environmental Health Research Consortium. https://www.canuedata.ca/canue/metadata.php

Ministry of Forests, Lands, Natural Resource Operations and Rural Development - GeoBC. (n.d.-a). *BC Transmission Lines—Data Catalogue* [Data Warehouse]. BC Data Catalogue. Retrieved September 23, 2020, from https://catalogue.data.gov.bc.ca/dataset/bc-transmission-lines

Ministry of Forests, Lands, Natural Resource Operations and Rural Development - GeoBC. (n.d.-b). *Digital Road Atlas (DRA)—Master Partially-Attributed Roads—Data Catalogue* [Data Warehouse]. BC Data Catalogue. Retrieved September 23, 2020, from https://catalogue.data.gov.bc.ca/dataset/digital-road-atlas-dra-master-partially-attributed-roads

Ministry of Forests, Lands, Natural Resource Operations and Rural Development - GeoBC. (n.d.-c). *Railway Track Line—Datasets—Data Catalogue* [Data Warehouse]. BC Data Catalogue. Retrieved September 23, 2020, from https://catalogue.data.gov.bc.ca/dataset/railway-track-line

Natural Resources Canada Lands and Minerals Sector. (2020). *Principal Mineral Areas, Producing Mines, and Oil and Gas Fields (900A) and Top 100 Exploration Projects in Canada* - [Data Warehouse]. Open Government Portal. https://open.canada.ca/data/en/dataset/000183ed-8864-42f0-ae43-c4313a860720

Natural Resources Canada Strategic Policy and Results Sector. (2020). *Location of mill facilities* [Data Warehouse]. Open Government Portal. https://open.canada.ca/data/en/dataset/46f1bb92-f936-4a56-a83b-c440db7c25d8

Pacific Climate Impacts Consortium. (2020). *Plan2Adapt* [Data Atlas]. Pacific Climate Impacts Consortium. https://services.pacificclimate.org/plan2adapt/app/

Potapov, P., Yaroshenko, A., Turubanova, S., Dubinin, M., Laestadius, L., Thies, C., Aksenov, D., Egorov, A., Yesipova, Y., Glushkov, I., Karpachevskiy, M., Kostikova, A., Manisha, A., Tsybikova, E., & Zhuravleva, I. (2008). Mapping the World's Intact Forest Landscapes by Remote Sensing. *Ecology and Society*, *13*(2). https://doi.org/10.5751/ES-02670-130251

Provincial Health Service Authority. (2020). *BC Community Health Data* [Data Atlas]. BC Community Health Atlas. http://communityhealth.phsa.ca/Home/AtlasFAQ

Statistics Canada. (2016). *2016 Census Profile of Dissemination Areas* [Canadian Census Analyser download]. http://dc.chass.utoronto.ca/census/

Wilderness Committee. (n.d.). *Kinder Morgan Trans Mountain Tar Sands Pipeline—Existing & Proposed Expansion* [Data Atlas]. Retrieved September 23, 2020, from https://www.arcgis.com/sharing/rest/content/items/b48aff5799614346970d8487cc0d8e00
